# Supplementary material for: Hexadecyltrimethylammonium hydroxide promotes electrocatalytic activity for the oxygen evolution reaction
Source: Commun Chem. 2020 Nov 4;3:154. doi: 10.1038/s42004-020-00406-w (PMC9814958; doi:10.1038/s42004-020-00406-w)
Supplement: Supplementary file 1 — Supplementary Information [file 42004_2020_406_MOESM1_ESM.docx]

**Hexadecyltrimethylammonium hydroxide promotes electrocatalytic activity for the oxygen evolution reaction**

**Yugan Gao, Chengqi Wu, Sen Yang, and Yiwei Tan***

*State Key Laboratory of Materials-Oriented Chemical Engineering, School of Chemistry and Chemical Engineering, Nanjing Tech University, Nanjing 211816, China, Email:* [*ytan@njtech.edu.cn*](mailto:ytan@njtech.edu.cn)

**Supplementary methods**

**Chemicals**

Ferric chloride hexahydrate (FeCl_3_·6H_2_O, >98%), sulfur powder (100 mesh, 99.5%), hydrazine hydrate (N_2_H_4_, 50–60%), ammonia borane (AB, 97%), hexadecyltrimethylammonium hydroxide solution (HTAH, 10 wt. % in water), terephthalic acid (BDC, >98%), and N,N-dimethylformamide (DMF, 99%) were commercially available from Sigma-Aldrich. Barium nitrate (Ba(NO_3_)_2_, 99.999%), strontium (II) nitrate (Sr(NO_3_)_2_, 99.5%), nickel(II) nitrate hexahydrate (Ni(NO_3_)_2_·6H_2_O, 99.99%), ferric(III) nitrate nonahydrate (Fe(NO_3_)_2_·9H_2_O, 99.99%), potassium hydroxide (KOH, >99%), ethylenediaminetetraacetic acid disodium dihydrate (EDTA-2Na, >99%), citric acid monohydrate (99.5%), ammonium hydroxide (25% NH_3_, 99%), and anhydrous ethanol (>99.5%) were purchased from Sinopharm Chemical Reagent Co., Ltd.. All reagents were used without any further purification. Nickel foam (NF) with a thickness of 1.5 mm was purchased from Ailantian Advanced Technology Materials Co., Ltd. and cut into equal parts (1.5 × 1.0 cm^2^). Before its usage, each piece of NF was sequentially pretreated with isopropanol, hydrochloric acid (0.1 M), and ethanol under ultrasonication to remove grease and the surface oxide layer. Ultrapure water (18.2 MΩ) produced with a Milli-Q purification system was used in the synthesis and electrochemical measurements.

**Characterization of materials**

Scanning electron microscopy (SEM) images and near-surface elemental mapping data were acquired using a Hitachi S-4800 field-emission scanning electron microscope equipped with an energy-dispersive X-ray spectroscopy (EDX) detector (Oxford) and operated at an accelerating voltage of 5 and 20 kV to investigate the morphology and near-surface chemical composition of the catalysts, respectively. Transmission electron microscopy (TEM) and high resolution TEM (HRTEM) micrographs were acquired using an FEI Tecnai G2 Spirit Bio TWIN and an FEI Tecnai G2 F20 S-Twin transmission electron microscope and operated at an accelerating voltage of 100 and 200 kV, respectively. Scanning TEM (STEM) micrographs and the corresponding EDX elemental maps were obtained in high-angle annular dark field (HAADF) mode using the same high resolution transmission electron microscope to obtain the bulk chemical composition of samples. The specimens for TEM observations were the powder samples or carefully scratched from the NF substrate and sonicated before dropping them onto 300 mesh carbon-coated copper grids. The metallic composition of the catalysts was also determined by inductively coupled plasma optical emission spectrometry (ICP-OES, PerkinElmer Avio 500, *λ* = 170–800 nm, *As* = 200 nm) after dissolving the sample in aqua regia. Atomic force microscopy (AFM) measurements were implemented by a Vecco Dimension 3100 SPM system. To obtain the phase and structure of samples, the X-ray diffraction (XRD) diffractograms were recorded using a Rigaku Smartlab diffractometer with a Cu Kα radiation (*λ* = 1.5406 Å) operating at 40 kV and 40 mA at a scanning rate of 0.0554°·s^–1^, and scanned in the Bragg‒Brentano mode in a step size of 0.02°. To evaluate the surface composition and elemental oxidation states of samples, X-ray photoelectron spectroscopy (XPS) measurements were carried out using a Kratos Axis Supra (Kratos Analytical, Shimadzu Group Company) spectrometer at 15 kV and 10 mA with a 180° double focusing hemispherical energy analyzer providing energy resolution of 0.1 eV, employing a monochromatized microfocused Al-Kα (*hv* = 1486.58 eV) X-ray source with 600 W X-ray power. Samples for XPS measurements were pretreated by repeated cycles of Ar^+^ ion sputtering to obtain clean sample surfaces. The binding energies (BEs) of the core levels were calibrated by setting the adventitious C 1s peak at 284.8 eV. Survey spectra of the samples in the BE range of 0–1000 eV and the core level spectra of the elemental signals were recorded at resolutions of 1 and 0.125 eV, respectively. The powder samples or scratched samples from the NF substrate were used as the specimen for XRD and XPS analyses after a cleaning treatment. Raman spectra were collected by using a Horiba-Jobin-Yvon LabRAM HR800 Raman spectrometer (excitation wavelength: 514 nm). The laser was focused on the samples with a confocal microscope equipped with a 50X long working distance objective (Olympus BX-30- LWD) for the visible lasers. The catalyst samples (ca. 7 mg of loose powder) were placed on a glass slide underneath the objective. For the acquisition of Raman spectra, the accumulation was collected at 60 s per scan for 5 scans with a 500 μm opening for the laser light. Nitrogen adsorption–desorption isotherms were measured by nitrogen physisorption at 77 K using a BELSORP mini II apparatus (Bel Inc.). To determine the specific surface areas (*S*_BET_) and pore size distributions of the samples, the Brunauer–Emmett–Teller (BET) method based on the adsorption data of the corresponding N_2_ isotherm in the relative pressure (*P*/*P*_0_) range from 0.04 to 0.20 and the Barrett-Joyner-Halenda (BJH) method by using nitrogen adsorption data, respectively. The samples were degassed under high vacuum (< 0.01 mbar) at 150 °C for at least 6 h prior to the measurements. The pH value of each solution containing a different HTAH concentration was recorded using a pH meter (SevenExcellence™ pH/mV, Mettler Toledo, Inc.) at room temperature.

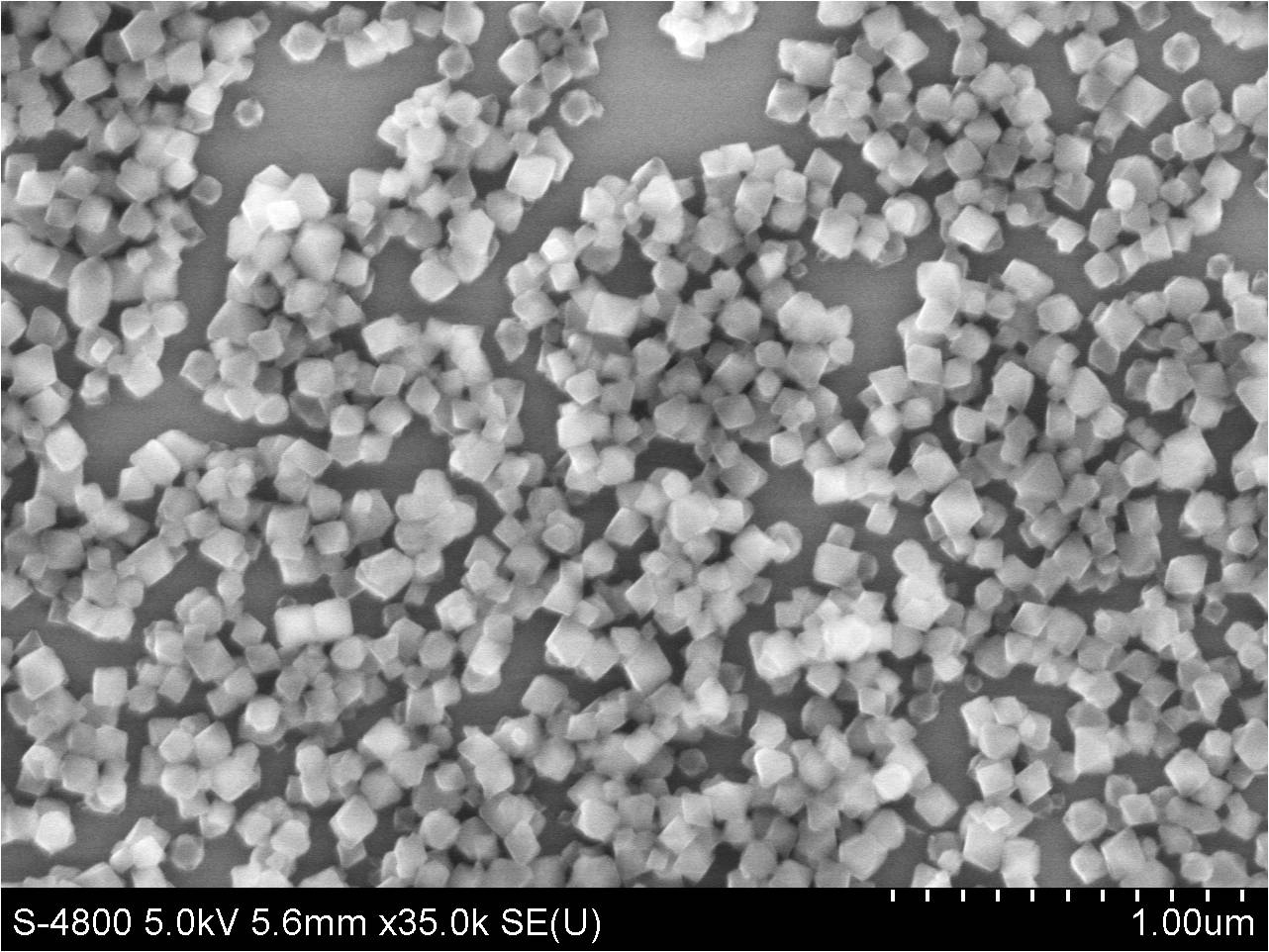


**Supplementary Fig. 1. Morphological and structural characterization of MIL-101 Fe.** (a) SEM image and (b) XRD pattern of the octahedral MIL-101 Fe precursor prepared according to the previous literature report.^1^

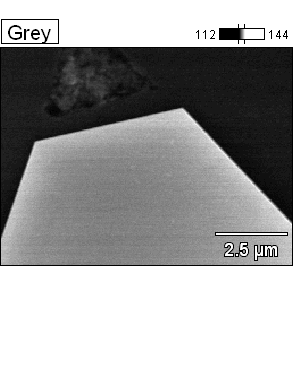

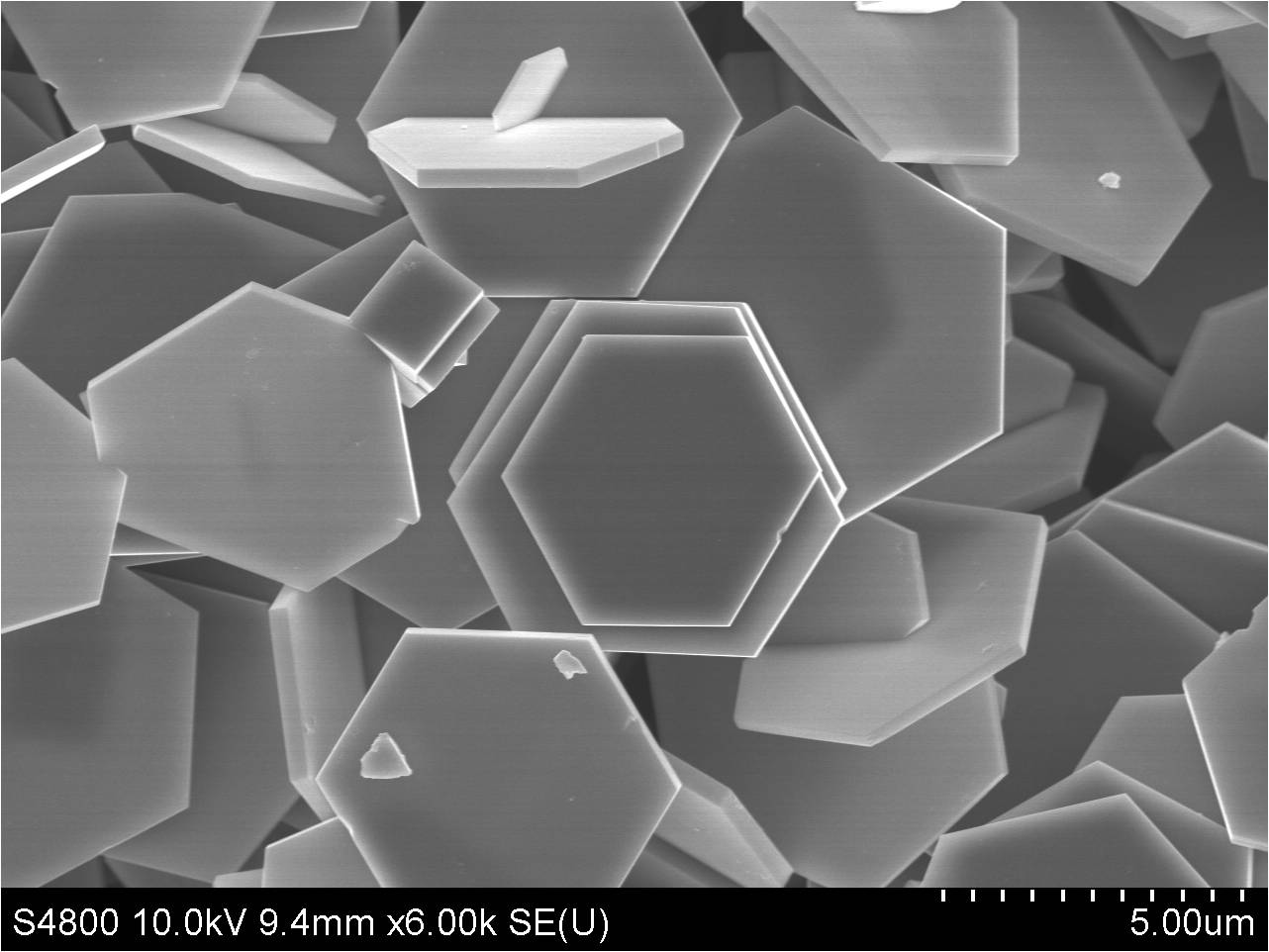

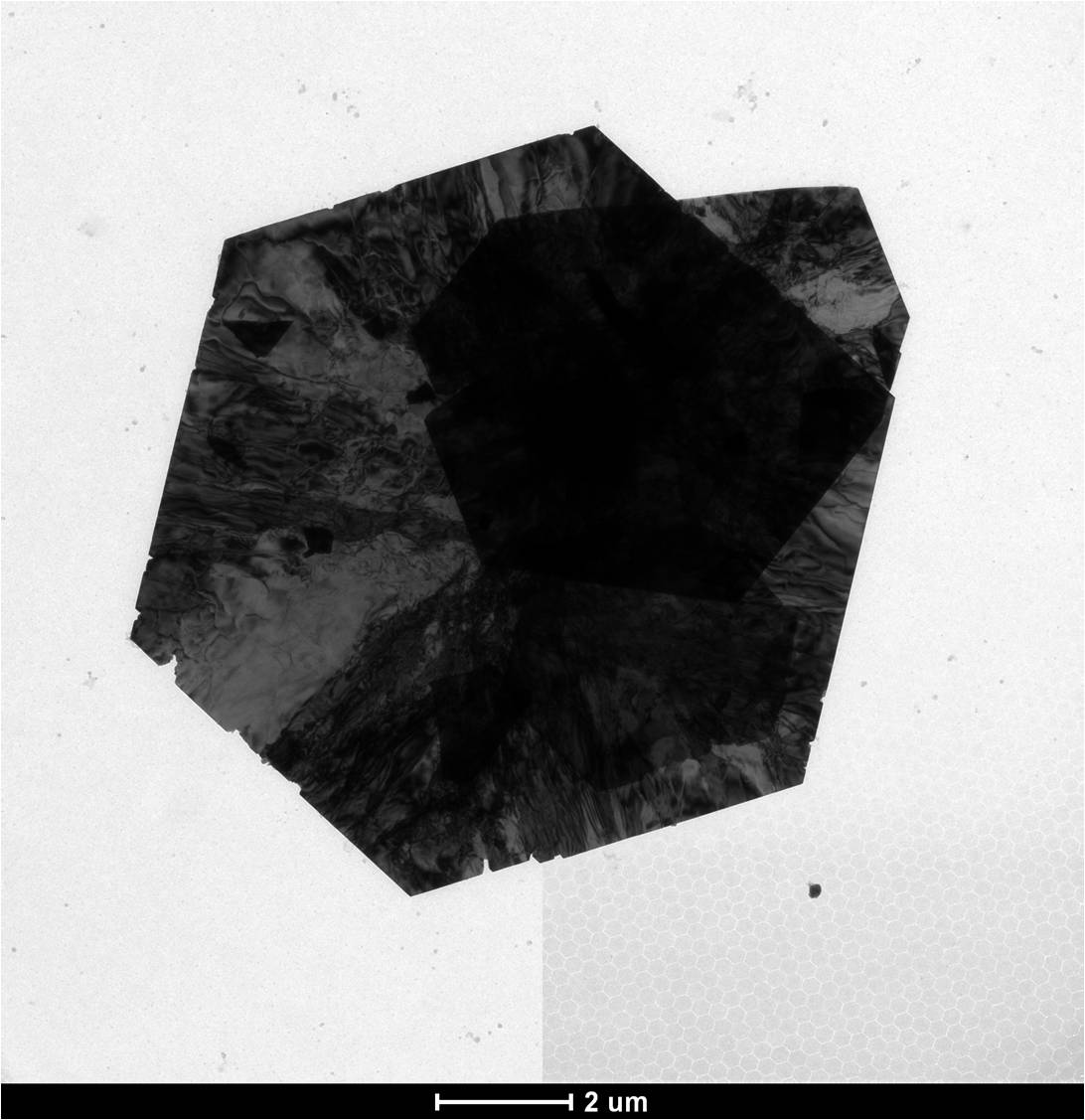

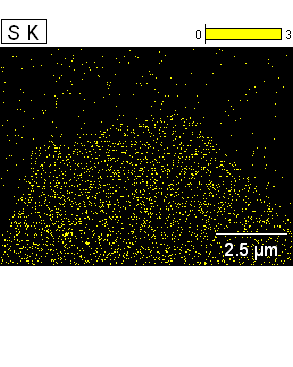

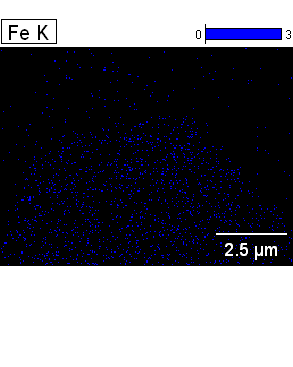


**Supplementary Fig. 2. Morphological, structural, and compositional characterization of iron sulfides microplatelets.** (a) SEM image, (b) TEM image, (c) XRD diffractogram, (d) SEM-EDX spectrum, and (e) typical SEM and (f and g) the corresponding SEM-EDX elemental mapping images of iron sulfides microplatelets. The intensities and positions for the pure Fe_7_S_8_ (red, JCPDF no. 24-0220), pyrite (blue, JCPDF no. 26-0801), and Fe_9_S_11_ (cyan, JCPDF no. 10-0437) references are provided as different colorful bars at the bottom according to the JCPDS database.

The SEM and TEM images in Supplementary Fig. 2 display the well-defined hexagonal microplatelets with an edge length of 1.0−3.5 μm and a thickness of 100−350 nm are formed after the sulfidization reaction of the octahedral MIL-101 Fe under the hydrothermal conditions (Supplementary Fig. 2a and b). The powder XRD pattern in Supplementary Fig. 2c reveals that all the diffraction peaks can be well assigned to three different phases, including Fe_7_S_8_ with a hexagonal structure (space group *P*31, *a* = 6.867 Å and *c* = 17.062 Å), FeS_2_ (pyrite) with a cubic structure (space group *Pa*3, *a* = 5.417 Å), and Fe_9_S_11_ with a rhombohedral structure (space group *R*3*m*, *a* = 3.470 Å and *c* = 34.500 Å), co-existed in the microplatelets. In particular, the TEM images show the large stresses evidenced by the bright and dark streaks, which are produced by the large lattice mismatch among the three different phases during crystal growth, existing in the microplatelets (Supplementary Fig. 2b). The composition of the as-synthesized microplatelets is further investigated by EDX spectrum and SEM-EDX elemental mapping. The results in Supplementary Fig. 2d exhibit that S and Fe peaks are found in addition to the Si peak from the Si substrate used for supporting the SEM specimen and the Fe/S atomic ratio is close to 46 : 54 by EDX quantitative analysis. As shown in Supplementary Fig. 2e−2g, the EDX elemental mapping images under the SEM mode reveal the homogenous distribution of S and Fe elements in the microplatelets, suggesting that the microplatelets are indeed comprised of S and Fe, which is consistent with the EDX spectrum and XRD pattern.

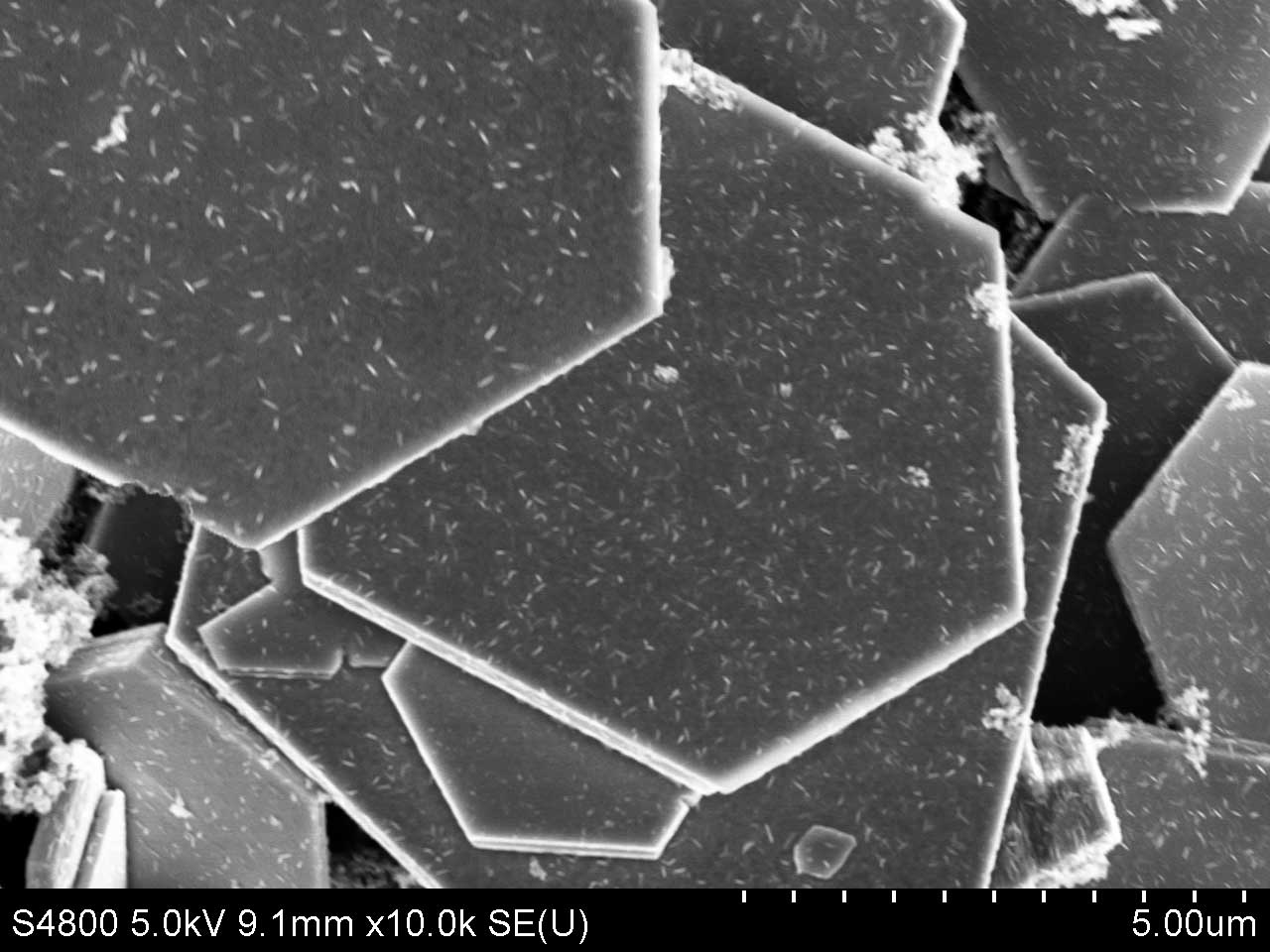


**Supplementary Fig. 3. Structural and compositional characterization of Ni-doped FeS_2_ (Fe_1−_*_y_*NiyS_2_) microplatelets.** (a) SEM image and (b) XRD diffractogram of the Fe_1−_*_y_*Ni*_y_*S_2_ microplatelets obtained after annealing of the hexagonal iron sulfides microplatelets supported on nickel foam (NF). The intensities and positions for the pure pyrite (blue, JCPDF no. 26-0801) and marcasite (orange, JCPDF no. 02-0908) references are given as different colorful bars at the bottom of panel (b) according to the JCPDS database.


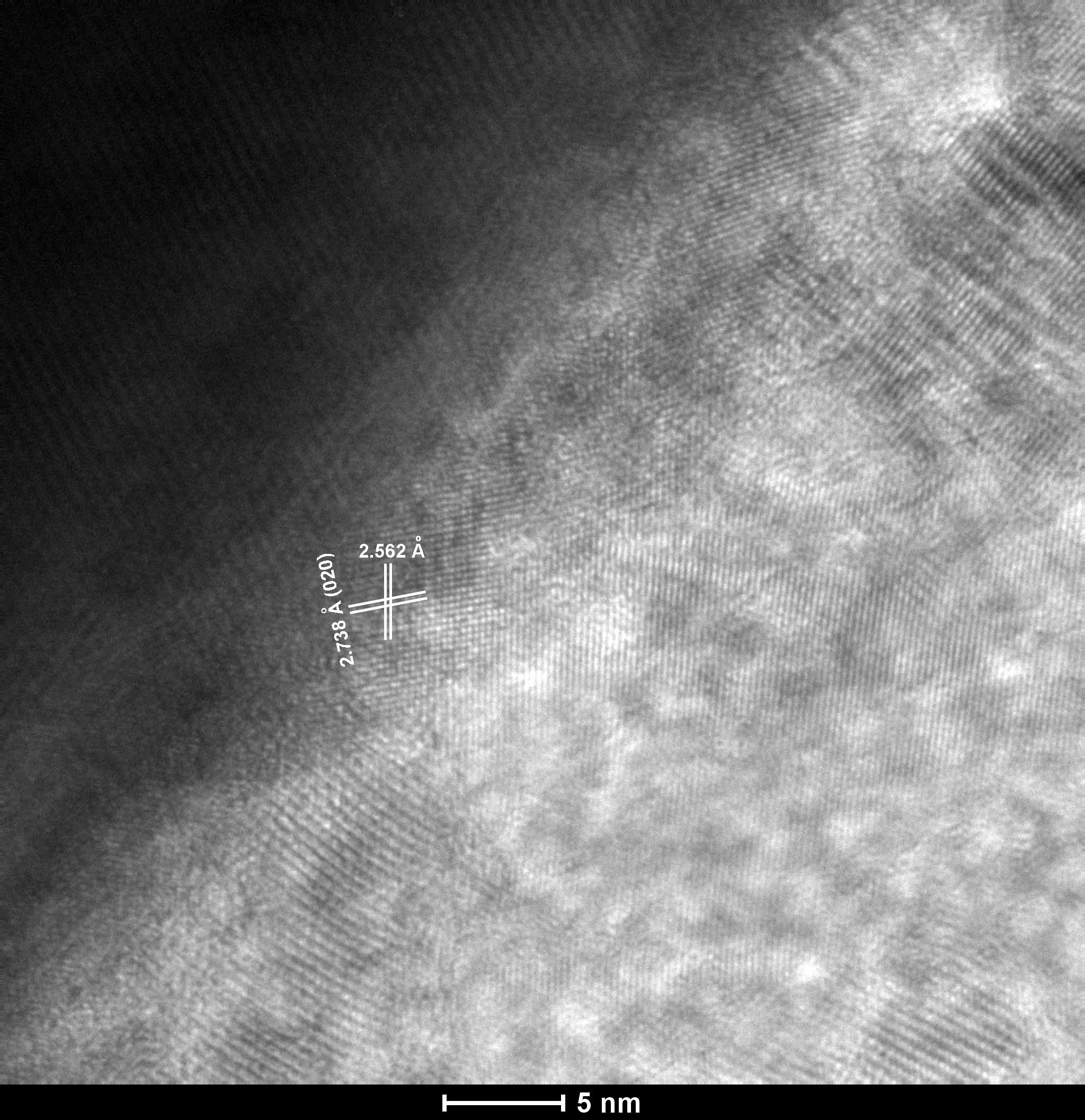

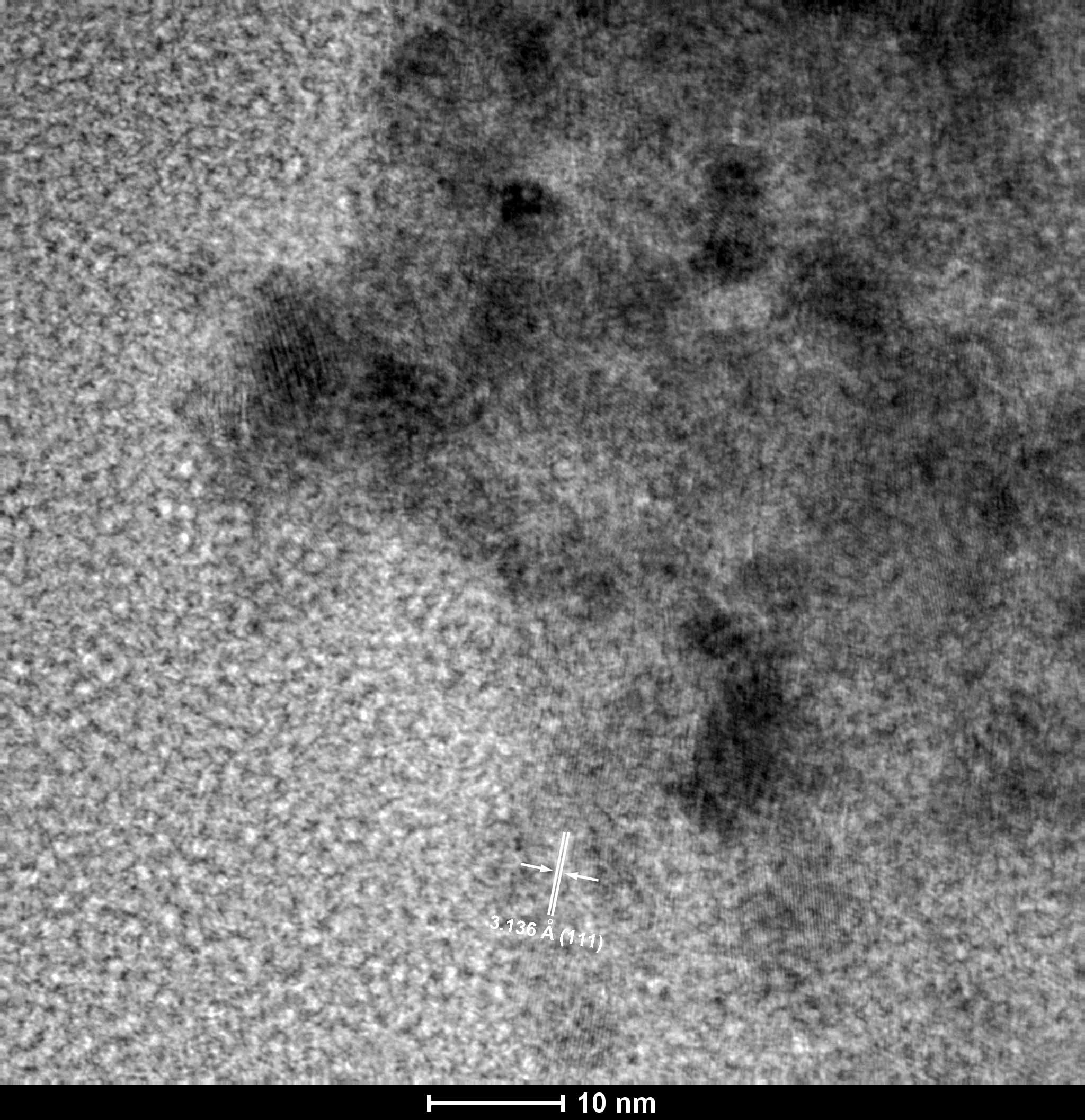

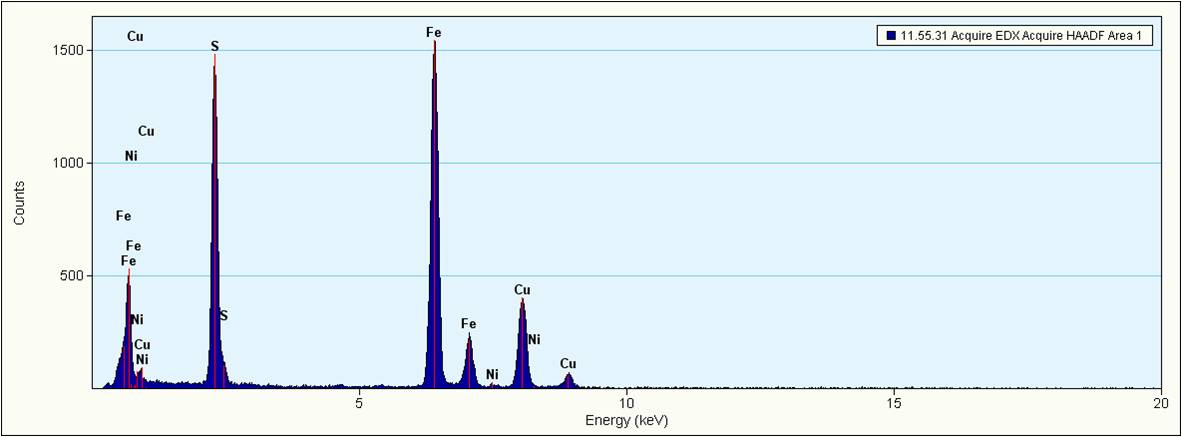


**Supplementary Fig. 4.** **Structural and compositional characterization of Fe_1−_*_y_*Ni*_y_*S_2_ microplatelets.** HRTEM images of the (a) marcasite and (b) pyrite phases and (c) EDX spectrum of the hexagonal Fe_1−_*_y_*Ni*_y_*S_2_ microplatelets. The HRTEM images were acquired in the fringe region of microplatelets.

The SEM image in Supplementary Fig. 3a shows that the surface of the annealed microplatelets (i.e., Fe_1−_*_y_*Ni*_y_*S_2_) becomes slightly rough with respect to the pristine microplatelets after annealing of the above-discussed iron sulfides microplatelets on NF at 350 ºC, while the hexagonal shape of the Fe_1−_*_y_*Ni*_y_*S_2_ microplatelets remains unchanged. The formation of Fe_1−_*_y_*Ni*_y_*S_2_ microplatelets whose crystal structure is different from that of the pristine microplatelets containing three different iron sulfide phases is supported by the powder XRD pattern in Supplementary Fig. 3b. It can be seen that Fe_1−_*_y_*Ni*_y_*S_2_ microplatelets show two sets of well-defined Bragg peaks, which can be perfectly indexed to the cubic pyrite-phase (*Pa*3 space group, *a* = 5.428 Å) as well as the orthorhombic marcasite-phase (*Pmnn* space group, *a* = 4.447 Å, *b* = 5.428 Å, and *c* = 3.389 Å), indicating the prominent phase transformation and alteration of the chemical stoichiometry of the resultant Fe_1−_*_y_*Ni*_y_*S_2_ after the annealing treatment. In particular, the high-temperature annealing of the iron sulfides microplatelets at 350 ºC enables the Ni atoms of NF to enter the crystal lattice of FeS_2_, resulting in the increased unit cell parameters of the two Fe_1−_*_y_*Ni*_y_*S_2_ phases compared to the corresponding pure FeS_2_ phase. Furthermore, the HRTEM observations also prove the presence of the pyrite-phase and marcasite-phase in Fe_1−_*_y_*Ni*_y_*S_2_ microplatelets (Supplementary Fig. 4a and b). The well-resolved, continuous lattice fringes in the Fe_1−_*_y_*Ni*_y_*S_2_ microplatelet domain with an interplanar spacing of 2.562 and 2.738 Å can be assigned to the unknown and (020) planes of the marcasite phase and suggest the single crystal feature of the Fe_1−_*_y_*Ni*_y_*S_2_ microplatelets, as illustrated in Supplementary Fig. 4a. Concurrently, the continuous lattice fringes with an interplanar spacing of 3.136 Å correspond well to the (111) planes of the pyrite phase (Supplementary Fig. 4b). The EDX spectrum in Supplementary Fig. 4c exhibits the presence of strong Fe and S signals as well as moderate Ni signals, verifying the composition of Fe_1−_*_y_*Ni*_y_*S_2_ microplatelets. The quantitative EDX and ICP-OES analysis consistently reveals that the average bulk Fe/Ni/S atomic ratio of Fe_1−_*_y_*Ni*_y_*S_2_ microplatelets is 0.9 : 0.1 : 2.


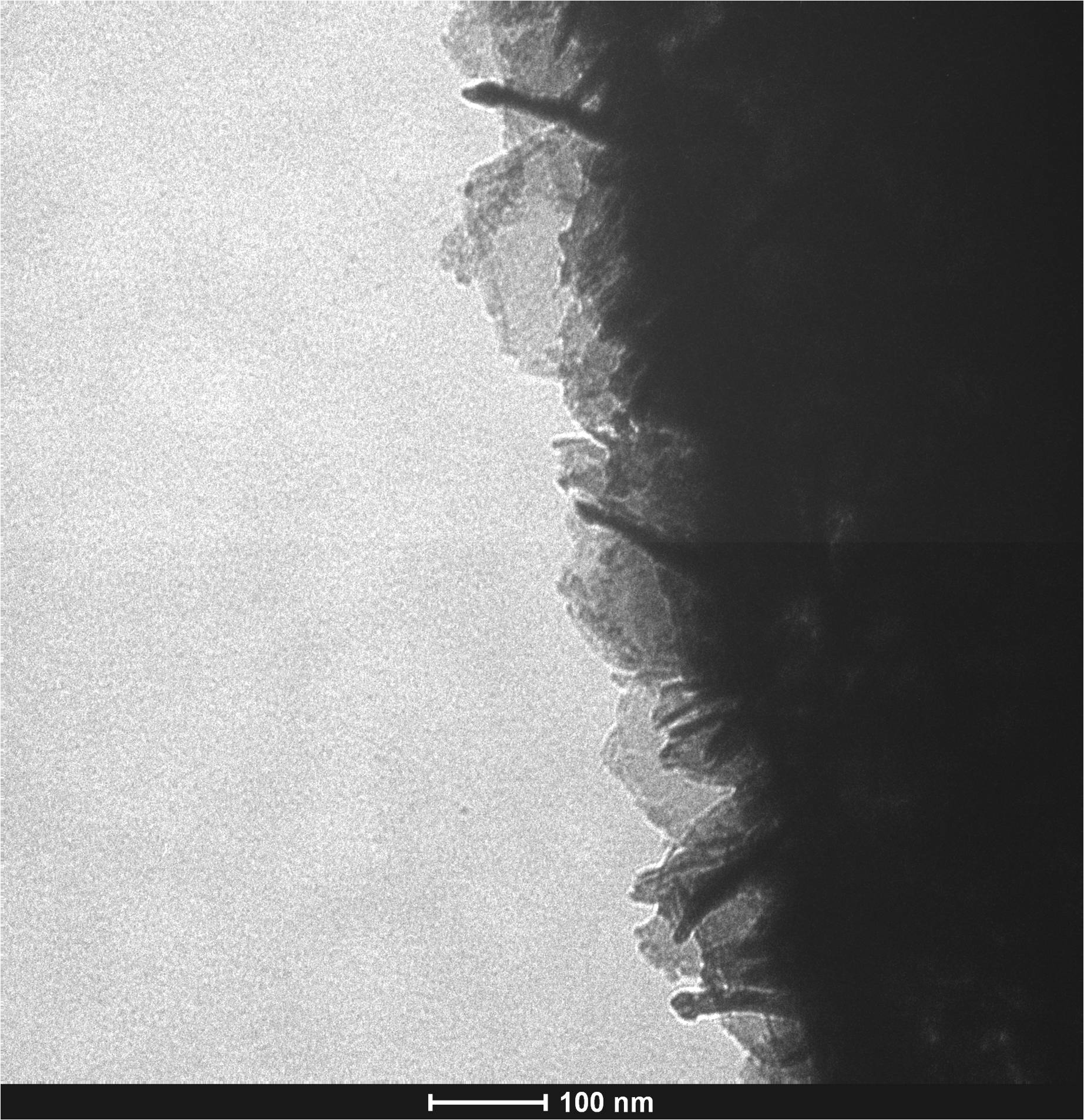

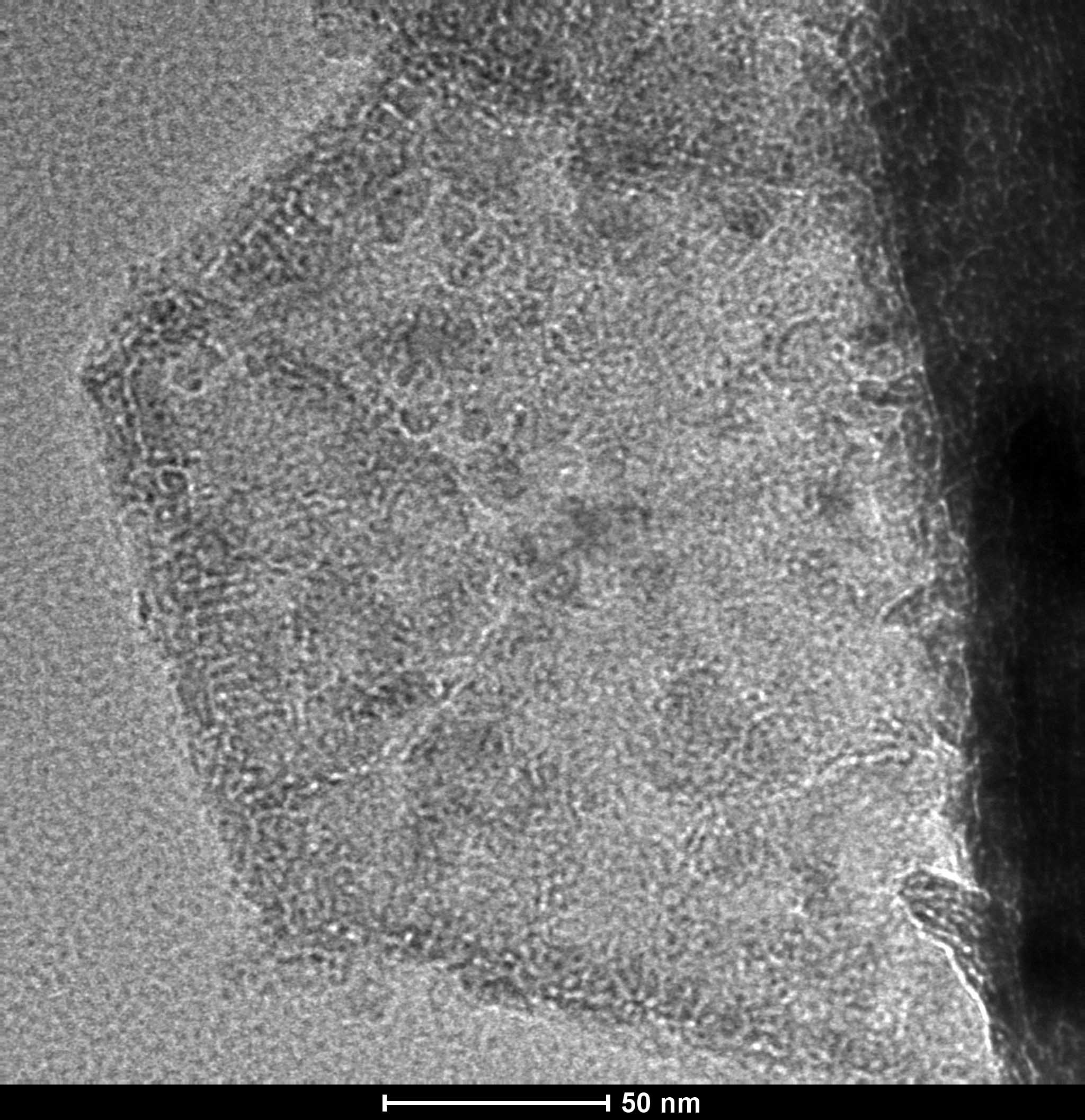


**Supplementary Fig. 5.** **TEM characterization of Fe_1−_*_x_*Ni*_x_*OOH nanoflakes.** (a) Low- and (b) high-magnification TEM images of the Fe_1−_*_x_*Ni*_x_*OOH nanoflakes grown on the Fe_1−_*_y_*Ni*_y_*S_2_@Fe_1−_*_x_*Ni*_x_*OOH microplatelets.


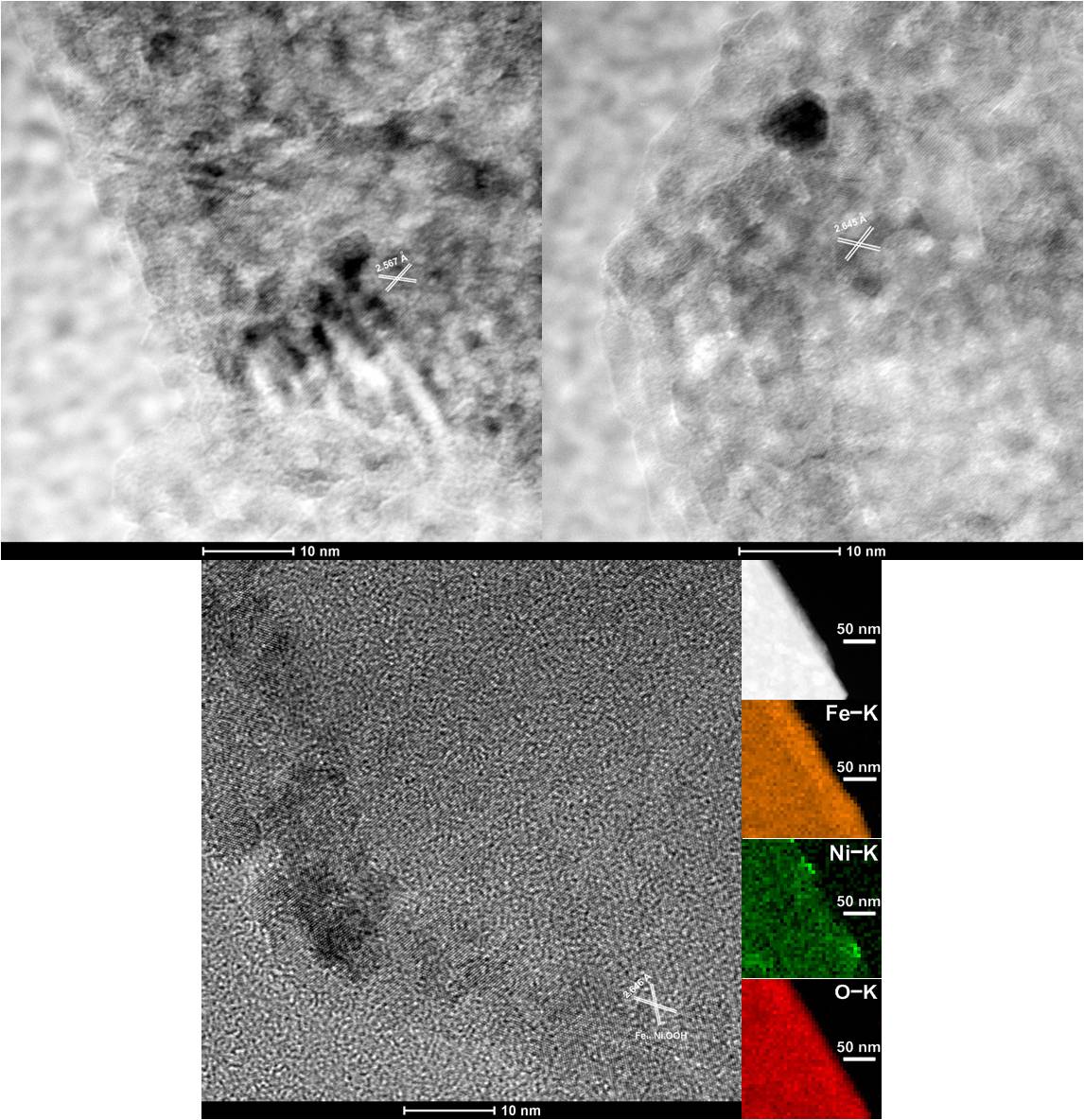


**Supplementary Fig. 6.** **HRTEM and elemental mapping images of the outer Fe_1−_*_x_*Ni*_x_*OOH nanoflakes of Fe_1−_*_y_*Ni*_y_*S_2_@Fe_1−_*_x_*Ni*_x_*OOH microplatelets.** HRTEM images show the nanoflakes consisting of (a and b) several stacked layered or (c) one layered Fe_1−_*_x_*Ni*_x_*OOH nanosheet, in which well-defined porous features can be observed. (d) HAADF-STEM image and the corresponding HAADF-STEM-EDX elemental mapping images of a monolayer Fe_1−_*_x_*Ni*_x_*OOH nanosheet.

`


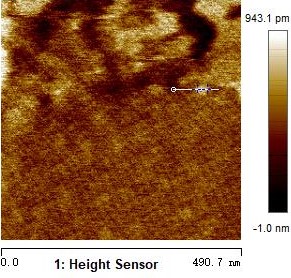

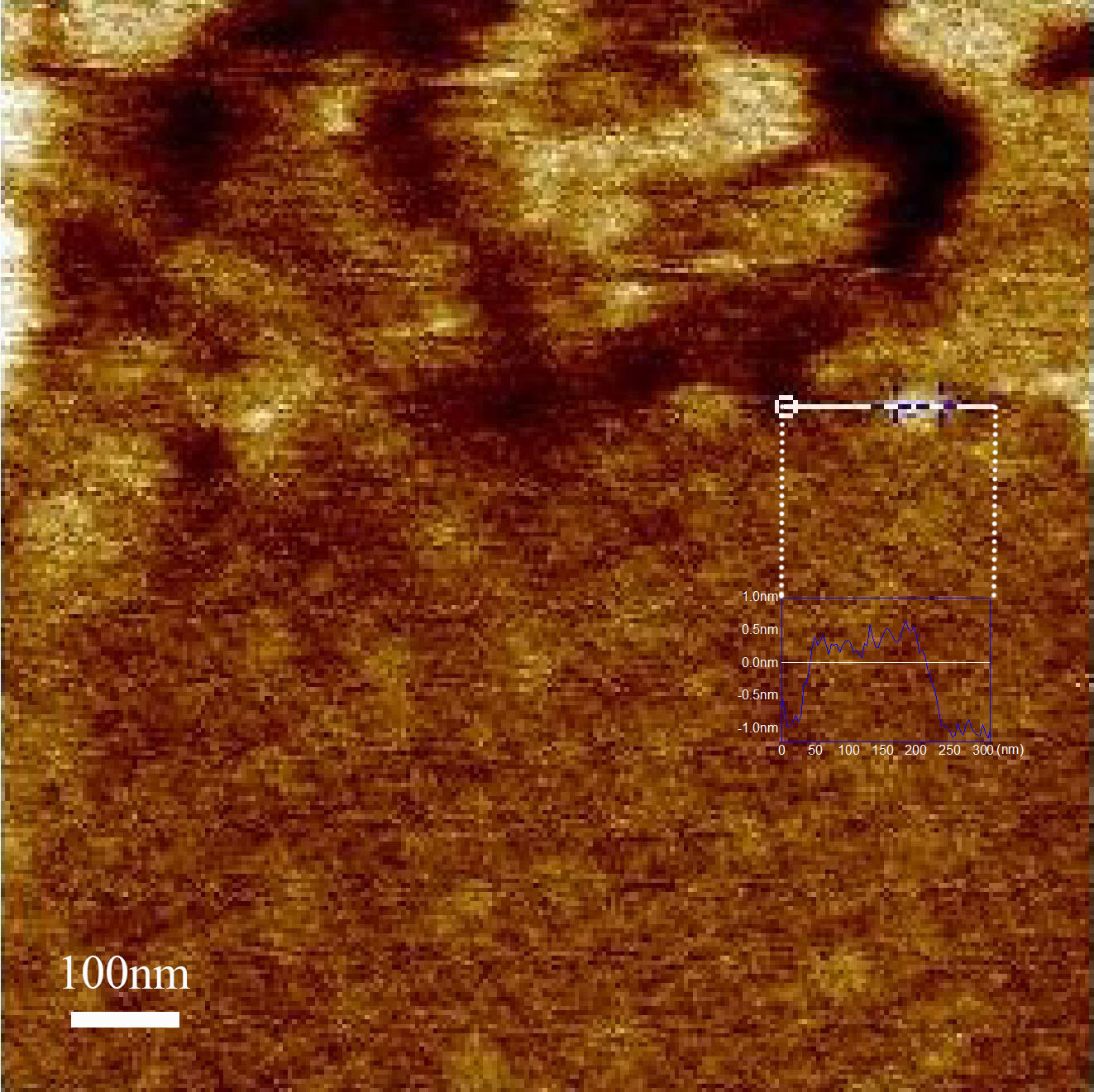


**Supplementary Fig. 7.** **AFM characterization of monolayer Fe_1−_*_x_*Ni*_x_*OOH nanosheets stripped from Fe_1−_*_y_*Ni*_y_*S_2_@Fe_1−_*_x_*Ni*_x_*OOH microplatelets.** (a) Low- and (b) high-magnification AFM images of a monolayer Fe_1−_*_x_*Ni*_x_*OOH nanosheet. The inset in panel (b) showing the height profiles to demonstrate the thickness of the Fe_1−_*_x_*Ni*_x_*OOH nanosheet.

**Supplementary Fig. 8.** **XPS characterization of Fe_1−_*_y_*Ni*_y_*S_2_@Fe_1−_*_x_*Ni*_x_*OOH microplatelets and the precursor Fe_1−_*_y_*Ni*_y_*S_2_ microplatelets.** (a) XPS Survey spectra, (b) Fe 2p, (c) Ni 2p, (d) S 2p, and (e) O 1s core-level spectra.

The survey spectra of both samples reveals the presence of Fe, Ni, S, and O elements in addition to the adventitious C species (Supplementary Fig. 8a), confirming the composition of the electrode active materials. The Fe 2p core-level spectrum of the precursor Fe_1−_*_y_*Ni*_y_*S microplatelets can be deconvoluted into two sets of peaks, of which one set consisting of two peaks at binding energy (BE) of 707.3 and 720.7 eV can be readily attributed to the Fe(II) 2p_3/2_ and Fe(II) 2p_1/2_ signals resulting from the Fe(II)−S bond,^2−4^ and the other consisting of two peaks at 709.0 and 722.8 eV corresponds to Fe(III) 2p_3/2_ and Fe(III) 2p_1/2_ of the Fe(III)−S and possible Fe(II)−O species (Supplementary Fig. 8b).^3,4^ However, that of the derived Fe_1−_*_y_*Ni*_y_*S_2_@Fe_1−_*_x_*Ni*_x_*OOH microplatelets is remarkably different, where the intensity of the peak at BE of 707.6 eV drastically decreases, indicating the fraction of the Fe(II) sulfide species greatly reduces on the Fe_1−_*_y_*Ni*_y_*S_2_@Fe_1−_*_x_*Ni*_x_*OOH surface, and a shift of the rest Fe 2p peaks towards the higher BEs, i.e., 711.0 and 723.7 eV for the Fe(II) 2p_3/2_ and Fe(II) 2p_1/2_ associated with FeSO_4_ species^4,5^ and 713.0 and 726.0 eV for the Fe(III) 2p_3/2_ and Fe(III) 2p_1/2_ associated with FeOOH, respectively,^4,6,7^ is observed after the AB-assisted electrochemical conditioning (Supplementary Fig. 8b). In addition, two shakeup satellite features with a very low intensity can be identified for the Fe_1−_*_y_*Ni*_y_*S_2_ (716.8 and 730.5 eV) and Fe_1−_*_y_*Ni*_y_*S_2_@Fe_1−_*_x_*Ni*_x_*OOH (716.6 and 733.7 eV).

The Ni 2p signal of the Fe_1−_*_y_*Ni*_y_*S_2_ can be fitted into two sets of doublets, one consisting of two peaks centered at 852.6 (Ni 2p_3/2_) and 870.6 eV (Ni 2p_1/2_) and the other consisting of two peaks at 854.6 (Ni 2p_3/2_) and 874.0 eV (2p_1/2_), which can be assigned to the species associated with Ni^δ+^ (0 < δ < 2) and Ni^ε+^ (2 < ε < 3) species, respectively (Supplementary Fig. 8c).^6,7^ In the case of the derived Fe_1−_*_y_*Ni*_y_*S_2_@Fe_1−_*_x_*Ni*_x_*OOH microplatelets, the fitting analysis of its Ni 2p core-level XPS spectrum demonstrates that the Ni species can be identified as Ni(II) species (853.7 (Ni 2p_3/2_) and 871.5 eV (Ni 2p_1/2_)) and Ni(III) species (855.9 (Ni 2p_3/2_) and 874.8 eV (Ni 2p_1/2_)) (Supplementary Fig. 8c).^7,8^ The Fe 2p and Ni 2p core-level spectra show substantial blue-shifts in the binding energies for Ni and Fe components in the Fe_1−_*_y_*Ni*_y_*S_2_@Fe_1−_*_x_*Ni*_x_*OOH with respect to those in the Fe_1−_*_y_*Ni*_y_*S_2_, being indicative of a significant increase in the overall oxidation state of these two metal atoms. In addition, two shakeup satellite peaks at 859.1 and 877.7 eV for the Fe_1−_*_y_*Ni*_y_*S_2_ and at 860.0 and 880.2 eV for the Fe_1−_*_y_*Ni*_y_*S_2_@Fe_1−_*_x_*Ni*_x_*OOH are associated with Ni 2p_3/2_ and Ni 2p_1/2_, respectively. It should be noted that the formation of the mixed oxidation states for Fe and Ni ions is extremely beneficial for the derived Fe_1−_*_y_*Ni*_y_*S_2_@Fe_1−_*_x_*Ni*_x_*OOH to improve its electronic conductivity.

The S 2p XPS spectra of both samples in Supplementary Fig. 8d shows that two BEs at 162.6 and 163.9−164.1 eV can be assigned to S 2p_3/2_ and S 2p_1/2_, respectively.^4,9^ In the S 2p region, the surface oxidized S species (SO_4_^2−^) is responsible for the additional peak at 168.3−168.6 eV, suggesting the presence of well-defined metallic sulfide.^3,4^ A large increase in SO_4_^2−^ peak intensity coincides with the observation of the significant surface oxidization after the AB-assisted electrochemical conditioning. The O 1s core-level spectra for the Fe_1−_*_y_*Ni*_y_*S_2_@Fe_1−_*_x_*Ni*_x_*OOH and Fe_1−_*_y_*Ni*_y_*S_2_ precursor can be fitted into four peaks centered at 529.7 and 530.1, 530.3 and 531.1, 531.4 and 532.0, and 532.3 and 532.9 eV by deconvolution, respectively, which can be tentatively assigned to the species associated with metal oxide (M(Fe, Ni)−O−M(Fe, Ni)) for the former two, metal hydroxides (M(Fe, Ni)−O−H), and adsorbed water (H_2_O), respectively (Supplementary Fig. 8e).^10−12^

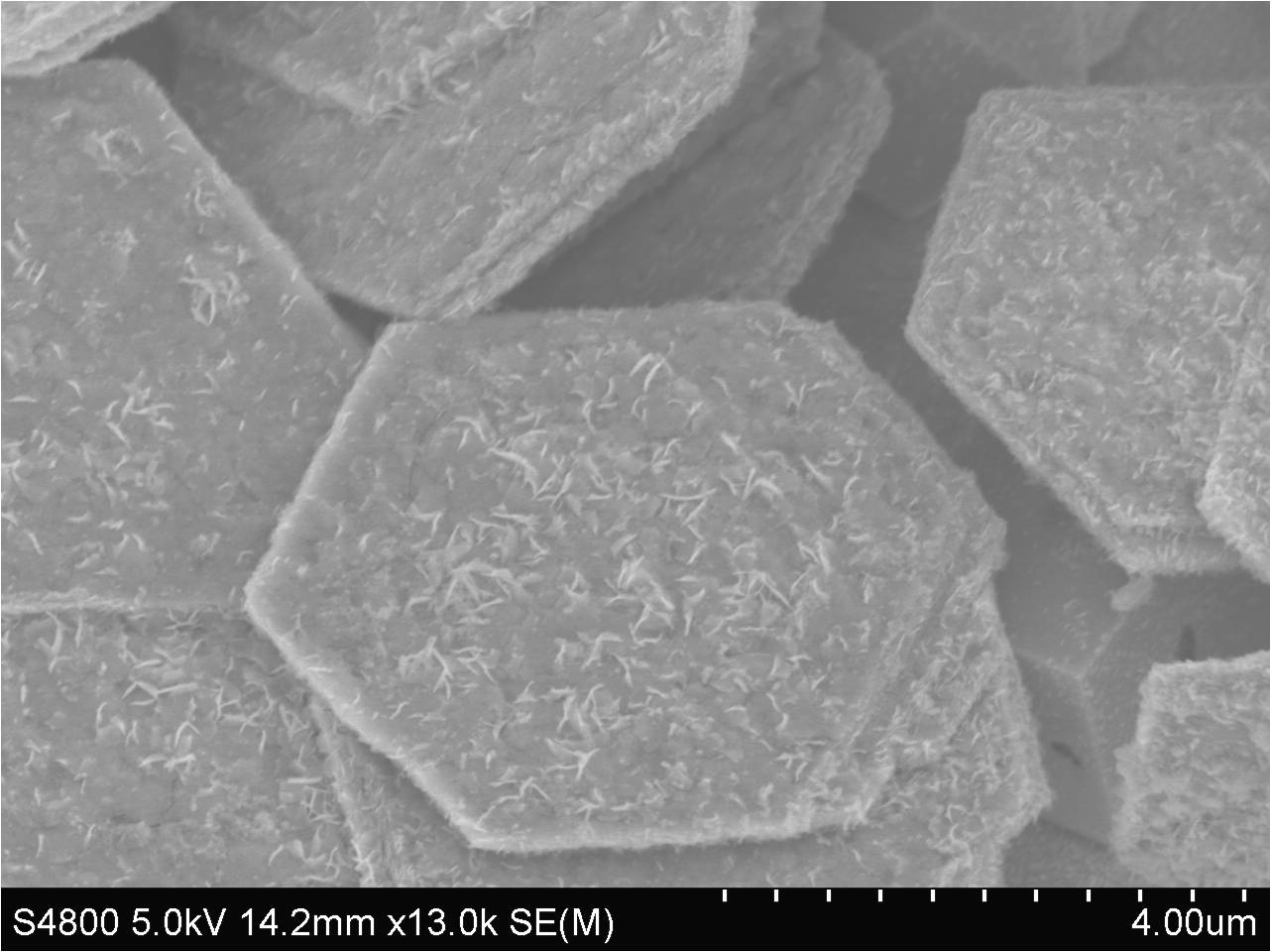


**Supplementary Fig. 9.** **Morphological and structural characterization of Fe_1−_*_y_*Ni*_y_*S_2_**-**ECC microplatelets.** (a) SEM image and (b) XRD pattern of the Fe_1−_*_y_*Ni*_y_*S_2_ microplatelets electrochemically conditioned in the absence of AB.

**Supplementary Fig. 10.** **The AB-assisted electrochemical conditioning process of Fe_1−_*_y_*Ni*_y_*S_2_ microplatelets.** Twenty continuous CV cyclings for the electrochemical conditioning of Fe_1−_*_y_*Ni*_y_*S_2_ microplatelets in the presence of 0.02 M AB to synthesize Fe_1−_*_y_*Ni*_y_*S_2_@Fe_1−_*_x_*Ni*_x_*OOH.

**Supplementary Fig. 11.** **Comparison of the OER electrocatalytic performance of Fe_1−_*_y_*Ni*_y_*S_2_-ECC/NF, Fe_1−_*_y_*Ni*_y_*S_2_/NF, and RuO_2_/NF reference in 1 M KOH and 0.98 M KOH + 0.02 M HTAH.** (a) The *iR*-corrected CV curves, (b and d) EIS, and (c and e) Tafel plots of (a−c) Fe_1−_*_y_*Ni*_y_*S_2_/NF electrochemically conditioned without AB and Fe_1−_*_y_*Ni*_y_*S_2_/NF and (d and e) RuO_2_/NF for water oxidation measured in 1 M OH^−^ solution prepared by 1 M KOH alone or 0.98 M KOH + 0.02 M HTAH. The CV curves and EIS spectra were recorded at a scan rate of 5 mV s^−1^ and a potential centered at 1.573 V_RHE_, respectively. Insets: (b) the Nyquist plots on a smaller scale (left) and fitted EEC used to model the electrode systems (right). (d) The EEC used to fit the EIS responses of the electrode system.

The fitted EEC in the right inset of Supplementary Fig. 11b is the two-time constant serial model (2 TS) including a series (electrolyte) resistance (*R*_s_), a resistance of the solution filling pores (*R*_1_) in the high frequency region, one constant phase element (CPE_1_) replacing the pore capacitance *C*_p_, charge transfer resistance (*R*_ct_) in the low frequency region, the other CPE_2_ replacing the interface capacitance *C*_int_ (i.e., *R*_s_, *R*_por_||CPE_1_ and *R*_ct_||CPE_2_). Meanwhile, there is an excellent agreement between the experimental data (symbols) and CNLS approximations (solid lines) when the 2 TS model is applied. The fitted *R*_s_ and *R*_ct_ of various samples are compared in Supplementary Table 1. A simple EEC in Supplementary Fig. 11d consists of a series resistance, *R*_s_, a charge transfer resistance, *R*_ct_, and a constant phase element (CPE).

**Supplementary Fig. 12.** **Evaluation of the ECSAs of the studied active materials in KOH alone.** Cyclic voltammograms of (a) the Fe_1−_*_y_*Ni*_y_*S_2_@Fe_1−_*_x_*Ni*_x_*OOH/NF, (b) Fe_1−_*_y_*Ni*_y_*S_2_/NF electrochemically conditioned without AB, and (c) Fe_1−_*_y_*Ni*_y_*S_2_/NF electrodes in 1 M KOH, which are used to estimate the double layer capacitance (*C*_dl_) in this electrolyte. Sweep rates at 10, 20, 40, 60, 80, and 100 mV s^−1^ were used. (d) Plots for the extraction of the *C*_dl_ value used for determining the ECSA of each electrode, which are obtained by Δ*j* = *j*_a_ − *j*_c_ at 1.05 V against scan rate (*v*).

**Supplementary Fig. 13.** **Evaluation of the ECSAs of the studied active materials in KOH + HTAH.** Cyclic voltammograms of (a) the Fe_1−_*_y_*Ni*_y_*S_2_@Fe_1−_*_x_*Ni*_x_*OOH/NF, (b) Fe_1−_*_y_*Ni*_y_*S_2_-ECC/NF electrochemically conditioned without AB, and (c) Fe_1−_*_y_*Ni*_y_*S_2_/NF electrodes in 0.98 M KOH + 0.02 M HTAH, which are used to estimate the double layer capacitance (*C*_dl_) in this mixed electrolyte. Sweep rates at 10, 20, 40, 60, 80, and 100 mV s^−1^ were used. (d) Plots for the extraction of the *C*_dl_ value used for determining the ECSA of each electrode, which are obtained by Δ*j* = *j*_a_ − *j*_c_ at 1.05 V against scan rate (*v*).

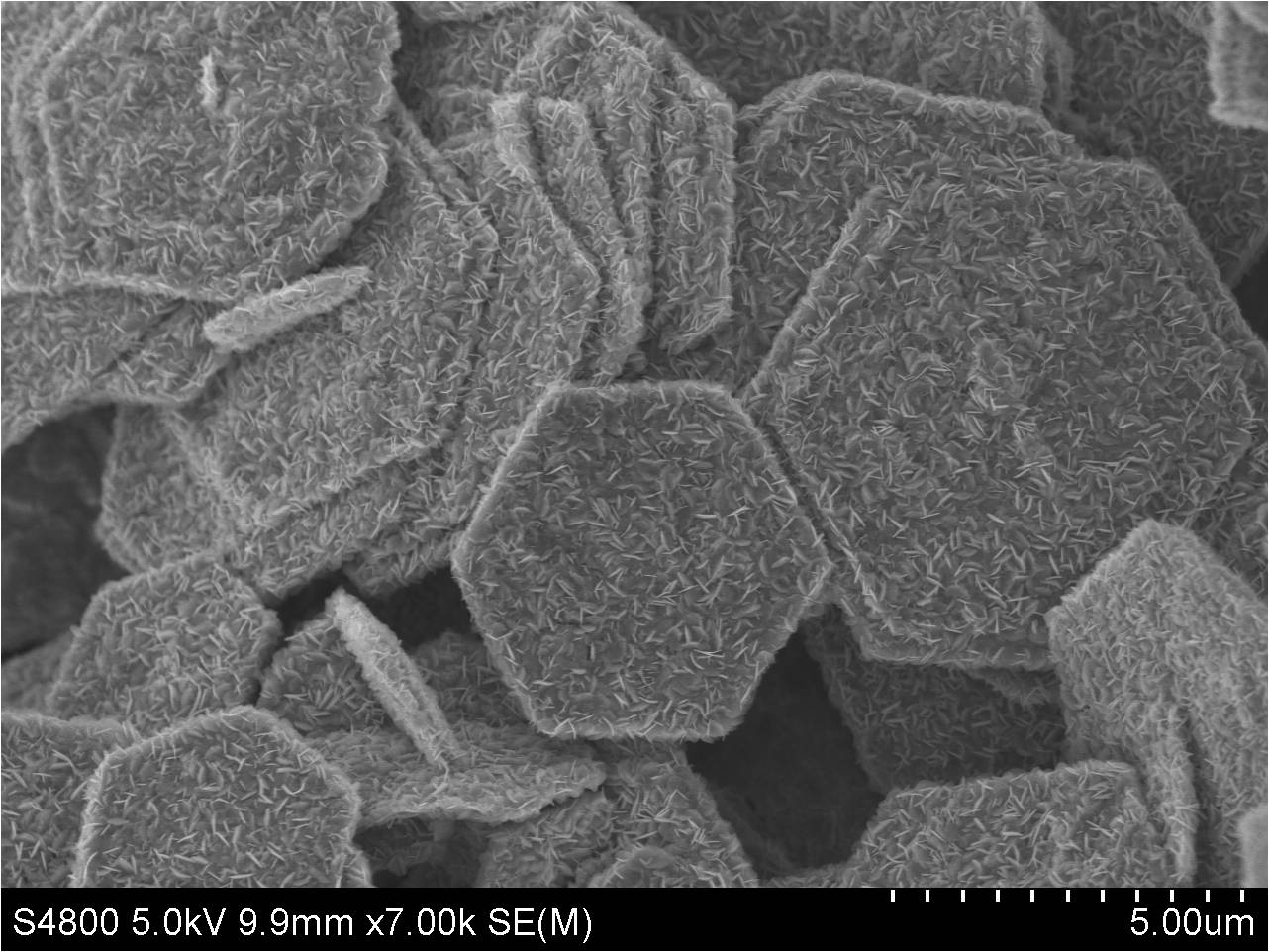

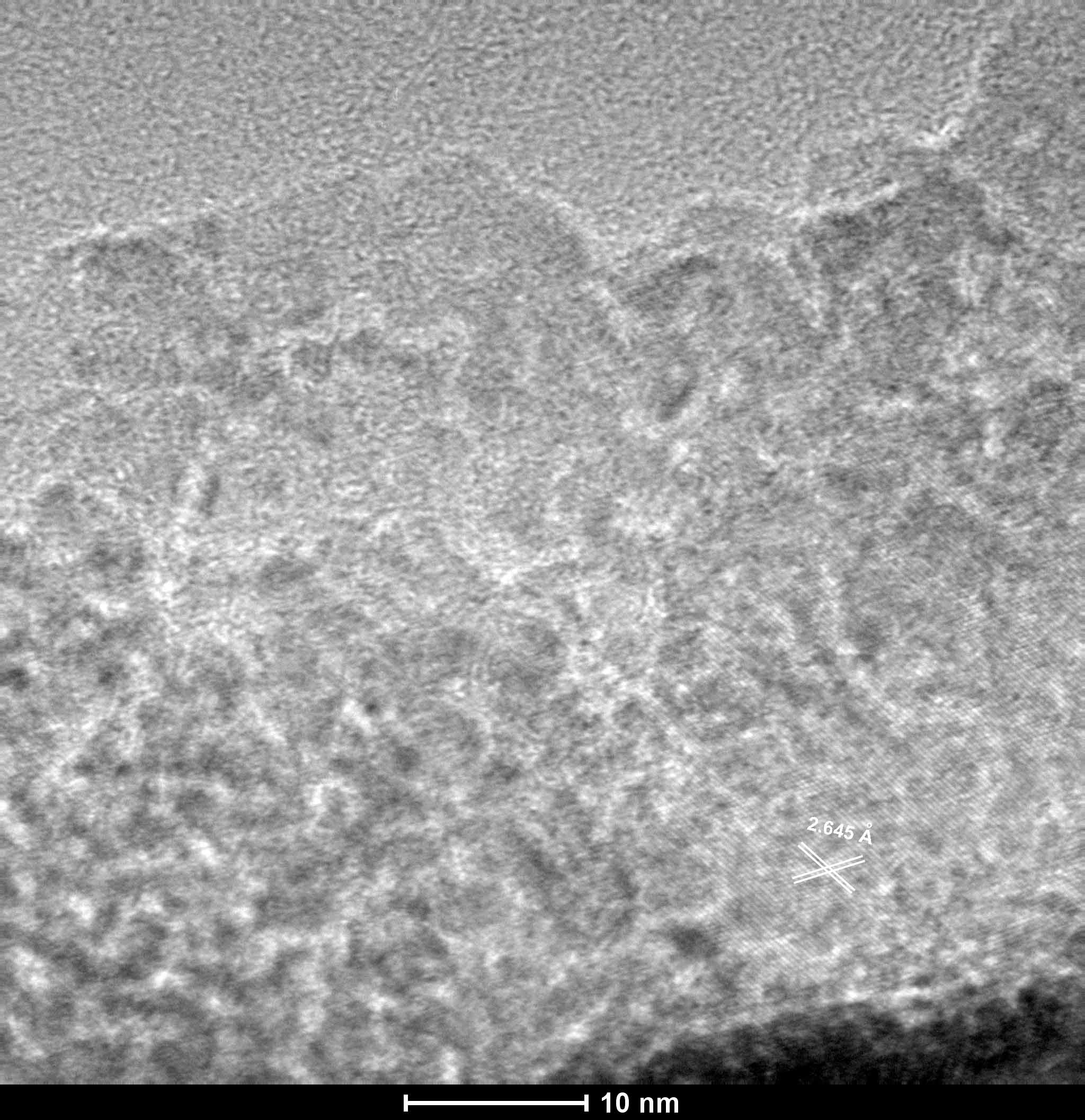


**Supplementary Fig. 14.** **Structural characterization of post-CP Fe_1−_*_y_*Ni*_y_*S_2_@Fe_1−_*_x_*Ni*_x_*OOH microplatelets.** (a) SEM image, (b) XRD diffractogram, and (c) HRTEM image of the Fe_1−_*_y_*Ni*_y_*S_2_@Fe_1−_*_x_*Ni*_x_*OOH obtained after the measurements of the OER. The intensities and positions for the pure pyrite (blue, JCPDF no. 26-0801), marcasite (orange, JCPDF no. 02-0908), and hydrohematite (cyan, JCPDF no. 02-0918) references are given as different colorful bars at the bottom of panel (b) according to the JCPDS database.

**Supplementary Fig. 15.** **Comparison of the OER electrocatalytic performance of Fe_1−_*_y_*Ni*_y_*S_2_@Fe_1−_*_x_*Ni*_x_*OOH/NF electrodes in different electrolytes without *iR* correction.** (a) The *iR*-uncorrected CV curves and (b) Tafel plots for Fe_1−_*_y_*Ni*_y_*S_2_@Fe_1−_*_x_*Ni*_x_*OOH/NF.

**Supplementary Table S1.** **The parameters for evaluating the electrocatalytic performance of Fe_1−_*_y_*Ni*_y_*S_2_@Fe_1−_*_x_*Ni*_x_*OOH/NF, Fe_1−_*_y_*Ni*_y_*S_2_-ECC/NF, Fe_1−_*_y_*Ni*_y_*S_2_/NF, SrBaNi_2_Fe_12_O_22_/GCD, and RuO_2_/NF catalysts towards the OER in KOH + HTAH and KOH alone.*^a^***

| Samples | *R*_s_ | *R*_1_ | *R*_ct_ | *C*_dl_ *^b^* | *η*_OER_ (mV) |
| --- | --- | --- | --- | --- | --- |
|  | (Ω cm^2^) | (Ω cm^2^) | (Ω cm^2^) | (mF cm^−2^) | (50 mA cm^−2^) |
| Fe_1−_*_y_*Ni*_y_*S_2_@Fe_1−_*_x_*Ni*_x_*OOH | 1.084 0.882 | 0.0203 0.0191 | 0.783 0.677 | 7.65 7.42 | 312 295 |
| Fe_1−_*_y_*Ni*_y_*S_2_-ECC | 1.782 1.749 | 0.0846 0.0725 | 1.475 1.267 | 3.44 3.21 | 350 333 |
| Fe_1−_*_y_*Ni*_y_*S_2_ | 2.357 2.145 | 0.0726 0.0724 | 2.701 2.458 | 2.07 2.12 | 385 361 |
| SrBaNi_2_Fe_12_O_22_ | 4.901 4.888 |  | 11.27 7.246 |  | 311 257 |
| RuO_2_ | 2.635 2.546 |  | 1.369 1.185 |  | 348 319 |

*^a^* The left and right column values for various active materials are obtained in 1 M KOH alone and 0.98 M KOH + 0.02 M HTAH, respectively. *^b^* The *C*_dl_ values were calculated according to the scan rate dependence of the charging current density, where the slope of the Δ*j* *vs*. scan rate (*v*) plot is twice *C*_dl_ (*i.e*., *C*_dl_ = Δ*j*/(2*v*).

**Supplementary Table S2.** **Comparison of electrocatalytic parameters of the Fe_1−_*_y_*Ni*_y_*S_2_@Fe_1−_*_x_*Ni*_x_*OOH/NF and SrBaNi_2_Fe_12_O_22_/GCD anodes and other Ni−Fe-based EC electrodes reported in the literature for the OER.**

| Catalyst/electrode | *η* (mV) at  *j* (mA cm^−2^) | Tafel slope  (mV dec^−1^) | Stability  (h at *j* (mA cm^−2^)) | Electrode  geometrical  area (cm^2^) | Electrolyte | Ref in  the text |
| --- | --- | --- | --- | --- | --- | --- |
| NiFeS/NF *^a^* | 189 at 100 | 119.4 | unstable | 1 | 1 M KOH | 33 |
| Ni-Fe-OH@Ni_3_S_2_/NF *^a^* | 300 at 100 | 93 | 50 at 100 | 0.09 *^c^* | 1 M KOH | 34 |
| NiFe LDH@NiCoP/NF *^a^* | ~350 at 100 | 48.6 | 100 at 10 | 1 | 1 M KOH | 35 |
| amorphous NiFe/NF *^a^* | ~370 at 100 | 28 *^b^* | 10 at 100 | N/A | 1 M KOH | 36 |
| Ni–Fe disulfide@oxyhydroxide/  GCD *^a^* | ~405 at 100 | 42.6 | 50 at 10 | 0.196 | 1 M KOH | 37 |
| NiFe(oxy)sulfide/GCD *^b^* | ~318 at 50 | 56.3 | 5.6 at 10 | 0.196 | 0.1 M KOH | 38 |
| Ni*_x_*Fe_1−_*_x_*Se_2_-DO/NF *^b^* | ~220 at 100 | 28 | 24 at 10 | 0.2 *^c^* | 1 M KOH | 39 |
| Ni–Fe–Se nanocages/GCD *^b^* | 270 at 100 | 24 | 22 at 5 | 0.196 | 1 M KOH | 40 |
| Fe–Ni–P–B–O nanocages/CFP *^b^* | 290 at 100 | 39 | 40 at 100 | N/A | 1 M KOH | 41 |
| Ni-Fe-O mesoporous NW/GC *^b^* | ~295 at 100 | 39 | 60 at 10 | 0.196 | 1 M KOH | 42 |
| Ni_5_P_4_/NiP_2_/NiFe LDH *^b^* | 243 at 100 | 46.6 | 72 at 10–200 | 1 | 1 M KOH | 43 |
| Fe foam@NiFe LDH *^b^* | 260 at 100 | 48.3 | 6000 at 1000 | 0.09 *^c^* | 1 M KOH | 44 |
| Fe_1−_*_y_*Ni*_y_*S_2_@Fe_1−_*_x_*Ni*_x_*OOH/NF *^a^* | 332 at 100 | 44.2 | 100 at 150 | 1 | 0.98 M KOH +  0.02 M HTAH | This work |
| Fe_1−_*_y_*Ni*_y_*S_2_@Fe_1−_*_x_*Ni*_x_*OOH/NF *^b^* | 265 at 100 | 35.5/36.9 | N/A | 1 | 0.98 M KOH +  0.02 M HTAH | This work |
| SrBaNi_2_Fe_12_O_22_/GCD *^b^* | 272 at 100 | 40.2/50.5 | 100 at 150 | 0.196 | 0.98 M KOH +  0.02 M HTA | This work |

*^a^* The results were not corrected for *iR* drop throughout the system. *^b^* The *iR* compensation was performed for the results. *^c^* Note that the electrocatalytic parameters were obtained on the electrodes with very small geometrical areas while the activities are extremely attracting and superior to our results. The tiny electrodes release less amounts of electrolyzed gas products and therefore can relieve the accumulation of gas bubbles around electrode to a great extent, facilitating the mass transport of reactants to access to the active sites of ECs. However, small electrodes have less significance for practical applications.

**Supplementary references**

1. Taylor-Pashow, K. M. L., Rocca, J. D., Xie, Z., Tran, S. & Lin, W. Postsynthetic modifications of iron-carboxylate nanoscale metal−organic frameworks for imaging and drug delivery. *J. Am. Chem. Soc*. **131**, 14261−14263 (2009).
2. Wadia, C., Wu, Y., Gul, S., Volkman, S. K., Guo, J. & Alivisatos, A. P. Surfactant-assisted hydrothermal synthesis of single phase pyrite FeS_2_ nanocrystals. *Chem. Mater*. **21**, 2568−2570 (2009).
3. Morrish, R., Silverstein, R. & Wolden, C. A. Synthesis of stoichiometric FeS_2_ through plasma-assisted sulfurization of Fe_2_O_3_ nanorods. *J. Am. Chem. Soc*. **134**, 17854−17857 (2012).
4. Seefeld, S., Limpinsel, M., Liu, Y., Farhi, N., Weber, A., Zhang, Y., Berry, N., Kwon, Y. J., Perkins, C. L., Hemminger, J. C., Wu, R. & Law, M. Iron pyrite thin films synthesized from an Fe(acac)_3_ ink. *J. Am. Chem. Soc*. **135**, 4412−4424 (2013).
5. Brion, D. Etude par spectroscopie de photoelectrons de la degradation superficielle de FeS_2_, CuFeS_2_, ZnS et PbS a l’air et dans l’eau. *Appl. Surf. Sci*. **5**, 133−152 (1980).
6. Liang, H., Gandi, A. N., Xia, C., Hedhili, M. N., Anjum, D. H., Schwingenschlögl, U. & Alshareef, H. N. Amorphous NiFe-OH/NiFeP electrocatalyst fabricated at low temperature for water oxidation applications. *ACS Energy Lett*. **2**, 1035−1042 (2017).
7. Sivanantham, A., Ganesan, P. & Shanmugam, S. Hierarchical NiCo_2_S_4_ nanowire arrays supported on Ni foam: an efficient and durable bifunctional electrocatalyst for oxygen and hydrogen evolution reactions. *Adv. Funct. Mater*. **26**, 4661−4672 (2016).
8. Wang, G., Wang, H., Chen, T. & Tan, Y. Ni_1−x_M_x_Se_2_ (M = Fe, Co, Cu) nanowires as anodes for ammonia-borane electrooxidation and the derived Ni_1−x_M_x_Se_2−y_–OOH ultrathin nanosheets as efficient electrocatalysts for oxygen evolution. *J. Mater. Chem. A* **7**, 16372−16386 (2019).
9. Faber, M. S., Dziedzic, R., Lukowski, M. A., Kaiser, N. S., Ding, Q. & Jin, S. High-performance electrocatalysis using metallic cobalt pyrite (CoS_2_) micro- and nanostructures. *J. Am. Chem. Soc*. **136**, 10053−10061 (2014).
10. Epling, W. S., Hoflund, G. B., Weaver, J. F., Tsubota, S. & Haruta, M. Surface characterization study of Au/α-Fe_2_O_3_ and Au/Co_3_O_4_ low-temperature CO oxidation catalysts. *J. Phys. Chem*. **100**, 9929−9934 (1996).
11. Torres, J., Perry, C. C., Bransfield, S. J. & Fairbrother, D. H. Low-temperature oxidation of nitrided iron surfaces. *J. Phys. Chem. B* **107**, 5558−5567 (2003).
12. Trotochaud, L., Ranney, J. K., Williams, K. N. & Boettcher, S. W. Solution-cast metal oxide thin film electrocatalysts for oxygen evolution. *J. Am. Chem. Soc*. **134**, 17253−17261 (2012).
